# Modeling health and well-being measures using ZIP code spatial neighborhood patterns

**DOI:** 10.1038/s41598-024-58157-w

**Published:** 2024-04-22

**Authors:** Abhi Jain, Michael LaValley, Kimberly Dukes, Kevin Lane, Michael Winter, Keith R. Spangler, Nina Cesare, Biqi Wang, Michael Rickles, Shariq Mohammed

**Affiliations:** 1 Department of Biostatistics, Boston University School of Public Health, Boston, 02118 USA; 2 Department of Environmental Health, Boston University School of Public Health, Boston, 02118 USA; 3 Biostatistics and Epidemiology Data Analytics Center, Boston University School of Public Health, Boston, 02118 USA; 4 Department of Medicine, University of Massachusetts Chan Medical School, Worcester, 01655 USA; 5 Sharecare, Research and Outcomes, Atlanta, 30305 USA; 6 Rafik B. Hariri Institute for Computing and Computational Science and Engineering, Boston University, Boston, 02215 USA

**Keywords:** Epidemiology, Statistics, Quality of life

## Abstract

Individual-level assessment of health and well-being permits analysis of community well-being and health risk evaluations across several dimensions of health. It also enables comparison and rankings of reported health and well-being for large geographical areas such as states, metropolitan areas, and counties. However, there is large variation in reported well-being within such large spatial units underscoring the importance of analyzing well-being at more granular levels, such as ZIP codes. In this paper, we address this problem by modeling well-being data to generate ZIP code tabulation area (ZCTA)-level rankings through spatially informed statistical modeling. We build regression models for individual-level overall well-being index and scores from five subscales (Physical, Financial, Social, Community, Purpose) using individual-level demographic characteristics as predictors while including a ZCTA-level spatial effect. The ZCTA neighborhood information is incorporated by using a graph Laplacian matrix; this enables estimation of the effect of a ZCTA on well-being using individual-level data from that ZCTA as well as by borrowing information from neighboring ZCTAs. We deploy our model on well-being data for the U.S. states of Massachusetts and Georgia. We find that our model can capture the effects of demographic features while also offering spatial effect estimates for all ZCTAs, including ones with no observations, under certain conditions. These spatial effect estimates provide community health and well-being rankings of ZCTAs, and our method can be deployed more generally to model other outcomes that are spatially dependent as well as data from other states or groups of states.

## Introduction

Well-being indices are useful tools that measure health and wellness across various dimensions. Individual-level responses can be aggregated to generate metrics that evaluate health and well-being for large and small geographical units, such as states, counties or ZIP Codes. From a community-health perspective, such aggregated metrics can be useful both at individual and population scales. That is, they can provide individuals and local decision-makers with valuable information and insights into specific aspects of local well-being that could potentially be reinforced and enhanced. Such information can be valuable to help inform policy and interventions.

There are several publicly available well-being indices that evaluate well-being at different geographic regions^1,2^. One example is the Robert Wood Johnson Foundation’s County Health Rankings, which uses data on more than thirty health and well-being measures to compute a composite score that serves as the basis of their ranking system^3^. Another example is the American Association of Retired Persons (AARP) Livability Index, which scores states, counties, ZIP Codes, and even neighborhoods on community quality of life^4^. Tobler’s First Law of Geography states that “everything is related to everything else, but near things are more related than distant things”^5^. However, counties are large spatial units and their formation, purpose, and governance varies widely both between and within states. They do not necessarily reflect functionally comparable units nationwide; neighboring counties may be vastly different sociodemographically and politically depending on the location. This is especially relevant in regions of the U.S. where counties are large in area and are thus potentially different to their neighboring counties in various aspects. Hence, assuming spatial similarity while evaluating county-level well-being^3,6^ might not be appropriate. However, ZIP Codes are substantially smaller spatial units that more closely resemble their neighbors, which makes the assumption of spatial similarity in them reasonable; it is also necessary to account for spatial correlations. ZIP Codes are often thought to represent individuals of similar demographics and socioeconomic status^7^, and ZIP Codes near each other likely have similar access to resources such as schools, healthcare infrastructure, food, and more. Thus well-being may also be similar across proximal ZIP Codes. Although the AARP Livability Index^4^ for ZIP Codes does include information from surrounding ZIP Codes or other neighborhoods, it does not explicitly use any spatial smoothing techniques or incorporate spatial neighborhood structure into a modeling framework. We seek to fill this gap by leveraging relevant spatial neighborhood structure of ZIP Code Tabulation Areas (ZCTAs) to assess ZCTA-level well-being.

In this paper, we will develop a statistical modeling framework that models individual-level well-being indices with individual-level covariates and a ZCTA-level effect. We employ graph Laplacian based regularization on the ZCTA effects to incorporate spatial neighborhood information into our model^8^. This allows for a spatially informed estimation of the effect of a ZCTA and its neighbors on individual health and well-being. This quantification will serve as the basis of generating ZCTA-level rankings. Neighborhood structures are often informed using boundaries of spatial units or Euclidean distance between pairs of units^9,10^. However, in our context of assessing well-being, we choose to use driving times between ZCTAs as it more accurately represents how people experience distance by travelling. Additionally, using driving times results in neighborhoods that are not uniform across the state since road and traffic conditions vary by location.

## Data

Partnering with the Boston University School of Public Health, Sharecare, a digital health company that offers consumers personalized health information, collects individual-level health data from across the country through digital surveys and Sharecare’s mobile app. These surveys pose 52 questions that comprise an overall score and scores for five subscales (Physical, Financial, Social, Community, and Purpose) to capture individual well-being and are defined as follows^11^. *Physical* is having good health and enough energy to get things done daily. *Financial* is managing economic life to reduce stress and increase security. *Social* is having supportive relationships and love in life. *Community* is liking where one lives, feeling safe, and having pride in their community. *Purpose* is liking what one does each day and being motivated to achieve one’s goals. Each year, a US county-level data collection strategy is deployed to capture individuals based on census demographic characteristics. Approximately 500,000 individuals, representing over 90% of the counties provide data annually. Over 80% of the sample is collected through Sharecare’s digital platforms and supplemental digital surveys are deployed to ensure that the data collection strategy targets are achieved. The WBI survey also provides an excellent test case for our spatial smoothing approach, as few other health indices aggregate patient-level data to different spatial resolutions.

We use the U.S. Census Bureau’s ZIP Code tabulation areas (ZCTA) from 2021 as our spatial units of interest. ZCTAs are similar to - but distinct from - ZIP Codes. The latter are codes defined by the US Postal Service (USPS) solely for the purpose of delineating mail delivery routes; they are neither polygonal spatial units nor intended for population analyses, and USPS does not publicly publish shapefiles of ZIP Code areas^12^. By contrast, ZCTAs are areal units defined by the US Census Bureau for the purpose of population analyses, and their shapefiles are publicly accessible. ZCTAs are intended to approximate the predominant ZIP Codes in a particular residential area^12^, but they are not always in 1:1 alignment. This is because ZCTAs are based on a *majority* of household ZIP Codes within each census block^12^; some households’ ZIP Codes may not match their respective ZCTAs. Although this introduces an unmeasurable amount of error (the Census Bureau cannot publish their point ZIP Code data due to privacy regulations), ZCTAs are nonetheless a useful areal approximation of an identifier that was not designed for population analyses. Overall, we identified 47/4443 (1.1%) and 880/116808 (0.8%) of Massachusetts and Georgia respondents whose reported ZIP Code was mapped to a different ZCTA, respectively. Lastly, ZCTAs can be non-contiguous and we evaluate the impact of non-contiguous ZCTAs on driving time assessment in Section 1 of the Supplementary Materials.

To inform our ZCTA-level spatial neighborhood pattern, we obtain driving times between ZCTA population centroids from OpenRouteService^13^ – details in the Methods section. However, since individuals report ZIP Codes as their place of residence and we use ZCTA geographies to perform analysis, we map ZIP Codes to ZCTAs using UDS Mapper’s Zip Code to ZCTA Crosswalk^14^ so each observation is mapped to a ZCTA.

**Figure 1:**
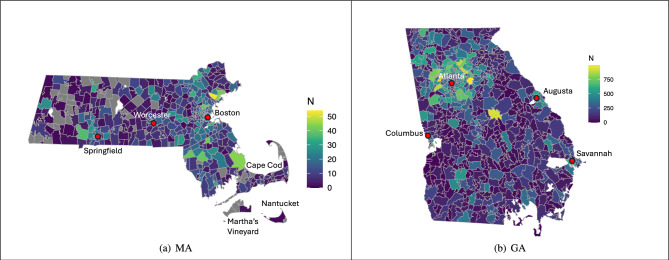
Choropleth maps of the total number of observations from each ZCTA in the Massachusetts and Georgia Sharecare WBI datasets. Grey ZCTAs are ones with zero observations and white areas are bodies of water or regions without assigned ZCTAs. Some of the important locations in Massachusetts and Georgia that are mentioned in this paper are also depicted. Note that the scales for the two states are different.

We use data from 2021, which has a sample size of nearly 500,000 for the entire United States, and select two states, Massachusetts and Georgia, as case studies. We select Massachusetts because it is one of the highest performing states in various well-being measures and also has a moderate sample size of 4443 observations, ranking 22nd out of the 50 states and Washington D.C. We select Georgia because it has the largest sample size at 116,808 observations and has the most comprehensive coverage across the state as compared to all other states. Note that 448 out of 539 (83%) ZCTAs are represented in Massachusetts, and 725 out of 751 (97%) ZCTAs in Georgia are represented. There is generally less coverage in central and western Massachusetts and higher coverage in Boston and suburban Boston (Fig. 1a); similarly, Georgia displays a comparable trend, with a large number of observations in the vicinity of Atlanta (Fig. 1b). That is, we observe the highest number of responses from around Boston and Atlanta—the two most populous cities in Massachusetts and Georgia, respectively.

Our main outcome variables are overall Well-Being Index (Overall WBI) and well-being across the five subscales (Physical, Financial, Social, Community, and Purpose). These scores are bounded between 0 and 100, with 100 representing the best possible score. We use individual-level demographic information such as gender, age, race, education, income, marital status, and urban/rural status as covariates.

## Results

We present a summary of the demographic characteristics of the sample from Massachusetts, Georgia, and the U.S as a whole in Table 1. In Massachusetts, over 61% of respondents are female and that number is over 72% in Georgia, with a national percentage of 64%. In Georgia, nearly 26% of respondents are Black and around 32% are respondents of color (defined as respondents who do not identify as White). Conversely, in Massachusetts, the percent of respondents of color is about 18%. Both Massachusetts and Georgia have around 36% of respondents who report an income over $\$100,000$ per year, and over 72% have at least a college education, with 43% of respondents in Georgia also having a post-graduate degree.

**Table 1:** Percentages of demographic characteristic for the entire United States (US), Massachusetts (MA), and Georgia (GA).

Variable	US	MA	GA
Gender			
Female	64.2	61.3	72.3
Age			
18–29	14.2	24.4	6.6
30–44	31.2	29.8	27.6
45–64	46.6	33.6	61.9
65+	7.9	12.2	4.0
Race			
White	73.4	82.2	67.5
Black	12.6	5.5	25.8
Hispanic/Latino	7.6	5.6	3.2
Asian	4.3	5.0	2.0
Other	2.1	1.8	1.4
Education			
<High school	1.3	2.9	0.5
High school	24.9	24.7	19.9
College	45.9	52.6	37.1
Post-graduate	27.9	19.8	42.5
Income			
<25K	6.6	14.6	4.4
25K–50K	15.8	18.6	17.8
50K–100K	35.7	30.4	41.6
100K+	42.0	36.4	36.3
Marital status			
Never married	21.1	37.5	14.0
Married	65.1	48.5	70.9
Divorced	13.8	14.0	15.1
Urban			
Urban	83.2	97.5	78.6

The sample size for the US is 495,783 while the sample sizes for MA and GA are 4443 and 116,808, respectively.

In Massachusetts, average scores for Overall WBI and the five subscales are generally higher in ZCTAs near Boston as well as some ZCTAs in Cape Cod and western Massachusetts (see Supplementary Figure S1 in Supplementary Materials). Conversely, ZCTAs in central Massachusetts near cities such as Worcester have the lowest average scores across several subscales. In Georgia, average scores for overall WBI and the subscales are highest in ZCTAs in the northern part of the state, while ZCTAs south of Atlanta and in the southwest part of the state have the lowest average scores across many of the subscales (see Supplementary Fig. S2 in Supplementary Materials). More details on the scores for WBI and the subscales are provided in Supplementary Table S1 of the Supplementary Materials.

We run the models by state, for Massachusetts and Georgia, with Overall WBI and scores from the five subscales as the outcome variables. The covariates include a set of individual-level demographic characteristics, i.e., age, sex, race, marital status, education, income, urban/rural status, and a ZCTA effect, $\alpha _s$ for ZCTA *s*. More details regarding the statistical framework are provided in the Methods section. Below we present the results from our analysis for both the states focusing on the regression parameter estimates of the demographic variables as well as the spatial effect estimates of ZCTAs.

### Massachusetts

As seen in Table 2, the effects of education and income are statistically significant for each subscale, with higher levels of education and income leading to higher scores, accounting for all other variables in our models. Compared to the reference group of less than a high school education, having a college degree (but not post-graduate degree) on average increases overall well-being by about 8.2 units, with a 95% confidence interval (CI) of (5.4, 11.0) units, holding all else constant. We see this same pattern emerge for each of the five subscales. Compared to those with an income less than $\$25,000$, individuals with an income of over $\$100,000$ have an increase in overall WBI of 13.7 units (95% CI: 12.0–15.4), on average, holding all else constant. The magnitude of the effect is larger for the Financial subscale with an increase of 27.0 (95% CI: 24.4–29.5) units. For financial and community subscales, the effect of age is also significant, with respondents aged 65 years or greater having better financial and community wellness compared to respondents aged 18-29 by 12.5 (95% CI: 9.6–15.3) and 7.2 (95% CI: 4.8–9.5) units, respectively, holding all else constant. Compared to individuals that have never married, married respondents have higher social and community well-being scores, with increases of about 5.3 (95% CI: 3.7–6.8) and 3.1 (95% CI: 1.5–4.6) units, respectively. Lastly, on average, participants residing in urban ZCTAs fare better than those residing in rural ZCTAs, with an effect of 17.5 units (95% CI: 14.5–20.6), holding all else constant. However, it is important to note that over 97% of respondents from Massachusetts are urban residents. Details on model fit are provided in Section 4 of the Supplementary Materials.

**Table 2:** Regression results for Overall WBI and the five subscales for Massachusetts.

	Overall WBI	Physical	Financial	Social	Community	Purpose
Gender (ref: Male)						
Female	−0.8 (−1.8, 0.1)	**−1.5** (−2.6, −0.4)	−1.1 (−2.6, 0.4)	0.5 (−0.8, 1.7)	−0.7 (−1.9, 0.5)	−0.5 (−1.7, 0.7)
Age (ref: 18–29)						
Age: 30–44	−0.4 (−1.8, 1.0)	**−1.9** (−3.5, −0.3)	−0.4 (−2.5, 1.6)	−1.5 (−3.2, 0.3)	**2.7** (1.0, 4.4)	−0.9 (−2.6, 0.9)
Age: 45–64	**1.8** (0.4, 3.3)	−0.8 (−2.4, 0.9)	**5.6** (3.4, 7.8)	−0.5 (−2.4, 1.3)	**5.5** (3.6, 7.3)	1.5 (−0.3, 3.4)
Age: 65+	**3.3** (1.4, 5.2)	−1.1 (−3.2, 1.1)	**12.5** (9.6, 15.3)	0.9 (−1.5, 3.3)	**7.2** (4.8, 9.5)	**3.3** (0.9, 5.6)
Race (ref: White)						
Black	0.6 (−1.5, 2.7)	2.2 (−0.1, 4.6)	−0.7 (−3.9, 2.4)	0.6 (−2.0, 3.3)	−1.7 (−4.3, 0.9)	0.1 (−2.5, 2.6)
Hispanic/Latino	**2.9** (0.8, 5.0)	**2.6** (0.2, 5.0)	**3.2** (0.1, 6.4)	**2.9** (0.3, 5.6)	2.3 (−0.3, 4.8)	**4.3** (1.7, 6.8)
Asian	**2.6** (0.4, 4.8)	**3.5** (1.0, 6.0)	**4.5** (1.2, 7.8)	0.8 (−2.0, 3.6)	1.9 (−0.8, 4.7)	1.3 (−1.4, 4.1)
Other race	−1.2 (−4.8, 2.3)	−0.7 (−4.8, 3.3)	0.0 (−5.3, 5.4)	−3.1 (−7.6, 1.4)	−1.6 (−6.0, 2.8)	−1.6 (−6.0, 2.8)
Marital (ref: Never married)						
Married	**1.7** (0.4, 2.9)	0.3 (−1.2, 1.7)	1.0 (−0.9, 2.9)	**5.3** (3.7, 6.8)	**3.1** (1.5, 4.6)	**1.8** (0.2, 3.3)
Other marital status	−0.3 (−1.9, 1.4)	−1.3 (−3.2, 0.6)	−1.3 (−3.8, 1.2)	0.5 (−1.6, 2.6)	1.1 (−1.0, 3.1)	0.7 (−1.3, 2.8)
Education (ref: < High school)						
High school	**7.1** (4.3, 9.9)	**8.6** (5.4, 11.8)	4.4 (−0.1, 8.9)	**4.0** (0.2, 7.7)	**6.2** (2.7, 9.7)	2.4 (−1.3, 6.1)
College	**8.2** (5.4, 11.0)	**10.3** (7.1, 13.5)	**4.8** (0.4, 9.3)	**4.2** (0.5, 7.9)	**6.9** (3.5, 10.4)	**4.0** (0.4, 7.7)
Post-graduate	**10.9** (7.9, 13.8)	**12.4** (9.1, 15.8)	**9.8** (5.1, 14.5)	**7.4** (3.5, 11.3)	**9.0** (5.3, 12.7)	**7.0** (3.2, 10.9)
Income (ref: < 25K)						
Income: 25–50K	**3.4** (1.7, 5.1)	**4.6** (2.7, 6.6)	**3.5** (1.0, 6.1)	**2.9** (0.7, 5.0)	1.8 (−0.3, 3.9)	2.0 (0.0, 4.1)
Income: 50–100K	**7.5** (5.9, 9.0)	**9.0** (7.2, 10.8)	**13.4** (11.0, 15.8)	**5.3** (3.3, 7.3)	**4.3** (2.3, 6.2)	**4.4** (2.4, 6.3)
Income: 100K+	**13.7** (12.0, 15.4)	**14.6** (12.7, 16.6)	**27.0** (24.4, 29.5)	**11.5** (9.4, 13.7)	**9.2** (7.1, 11.3)	**9.3** (7.2, 11.4)
Urban (ref: Rural)						
Urban	**17.5** (14.5, 20.6)	**17.2** (13.7, 20.6)	**8.5** (3.3, 13.7)	**13.1** (8.7, 17.4)	**18.3** (14.5, 22.1)	**10.5** (5.8, 15.2)

The reference groups for the categorical variables are given in parentheses next to the variable name. Coefficient estimates in bold are statistically significant at the 5% level and 95% confidence intervals are in parentheses.

**Table 3:** Regression results for Overall WBI and the five subscales for Georgia.

	Overall WBI	Physical	Financial	Social	Community	Purpose
Gender (ref: Male)						
Female	**–1.1** (−1.3, −0.9)	**–1.1** (−1.3, −0.9)	**–0.9** (−1.2, −0.6)	**–0.7** (−0.9, −0.5)	**–1.1** (−1.4, −0.9)	**–1.2** (−1.5, −1.0)
Age (ref: 18–29)						
Age: 30–44	**0.4** (0.0, 0.8)	**−0.4** (−0.8, 0.0)	**2.5** (1.9, 3.1)	**–1.2** (−1.7, −0.8)	**1.8** (1.3, 2.2)	0.1 (−0.3, 0.6)
Age: 45–64	**3.7** (3.3, 4.0)	**1.8** (1.4, 2.2)	**9.2** (8.7, 9.8)	**1.7** (1.2, 2.1)	**5.1** (4.6, 5.5)	**3.9** (3.5, 4.4)
Age: 65+	**8.8** (8.3, 9.4)	**5.7** (5.1, 6.3)	**18.1** (17.3, 18.9)	**7.2** (6.5, 7.9)	**10.1** (9.5, 10.8)	**10.1** (9.5, 10.8)
Race (ref: White)						
Black	**–0.8** (−1.0, −0.5)	**–0.8** (−1.0, −0.5)	**–2.8** (−3.1, −2.5)	**0.4** (0.2, 0.7)	−0.2 (−0.4, 0.1)	**–1.0** (−1.3, −0.8)
Hispanic/Latino	**1.2** (0.7, 1.7)	**1.8** (1.3, 2.3)	−0.3 (−1.0, 0.4)	**1.5** (1.0, 2.1)	**0.8** (0.2, 1.3)	**1.5** (0.9, 2.1)
Asian	**4.0** (3.4, 4.6)	**5.7** (5.1, 6.4)	**5.1** (4.2, 6.0)	**2.1** (1.4, 2.9)	**2.7** (2.0, 3.5)	**2.4** (1.7, 3.2)
Other race	0.1 (−0.6, 0.8)	**1.0** (0.2, 1.7)	**–2.6** (−3.6, −1.5)	0.6 (−0.2, 1.5)	−0.6 (−1.5, 0.2)	0.6 (−0.3, 1.5)
Marital (ref: Never married)						
Married	**3.2** (2.9, 3.5)	**1.8** (1.4, 2.1)	**1.9** (1.5, 2.4)	**7.4** (7.0, 7.7)	**4.4** (4.1, 4.7)	**2.7** (2.3, 3.0)
Other marital status	**1.5** (1.2, 1.9)	**2.1** (1.7, 2.4)	−0.4 (−0.9, 0.1)	**1.0** (0.6, 1.4)	**2.0** (1.6, 2.4)	**1.6** (1.2, 2.0)
Education (ref: < High school)						
High school	**4.5** (3.4, 5.6)	**5.0** (3.8, 6.3)	**3.5** (1.7, 5.2)	**4.5** (3.1, 5.9)	**3.2** (1.8, 4.5)	**3.6** (2.2, 5.1)
College	**5.4** (4.2, 6.5)	**6.3** (5.1, 7.5)	**3.6** (1.8, 5.3)	**4.8** (3.4, 6.2)	**3.7** (2.3, 5.1)	**5.0** (3.5, 6.4)
Post-graduate	**7.1** (6.0, 8.3)	**7.5** (6.3, 8.8)	**5.8** (4.0, 7.6)	**6.7** (5.3, 8.1)	**5.9** (4.5, 7.3)	**7.7** (6.2, 9.1)
Income (ref: < 25K)						
Income: 25–50K	**2.5** (2.1, 3.0)	**2.4** (1.9, 2.9)	**5.8** (5.2, 6.5)	**2.0** (1.4, 2.5)	**1.9** (1.4, 2.4)	**1.9** (1.3, 2.4)
Income: 50–100K	**5.4** (5.0, 5.9)	**4.5** (4.1, 5.0)	**13.7** (13.0, 14.3)	**4.4** (3.9, 4.9)	**4.7** (4.1, 5.2)	**4.0** (3.4, 4.5)
Income: 100K+	**8.7** (8.3, 9.2)	**7.5** (7.0, 8.1)	**21.5** (20.8, 22.2)	**7.3** (6.7, 7.9)	**7.1 **(6.6, 7.7)	**6.5** (5.9, 7.1)
Urban (ref: Rural)						
Urban	**1.9** (1.1, 2.6)	**1.7** (0.9, 2.5)	−0.4 (−1.3, 0.4)	**2.0** (1.1, 2.9)	**2.1** (1.3, 3.0)	**–1.0** (−1.8, −0.3)

Reference groups for the categorical variables are given in parentheses next to the variable name. Coefficient estimates in bold are statistically significant at the 5% level and 95% confidence intervals are in parentheses.

**Figure 2:**
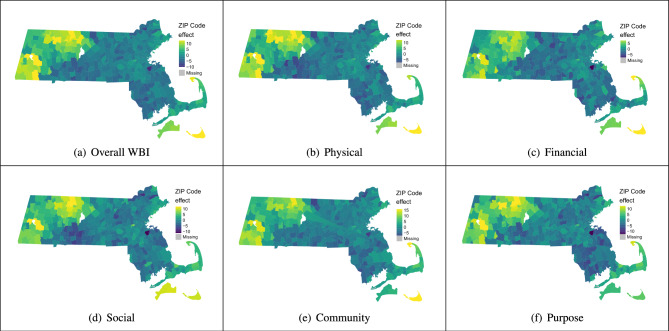
Choropleth maps of estimated ZCTA effects for overall WBI and the five subscales for Massachusetts. These ZCTA effect estimates represent the effect belonging to a ZCTA has on WBI and the five subscales after controlling for individual-level demographic covariates. To facilitate improved visualization, we threshold the estimated spatial effects exceeding three standard deviations from the mean and make them equal to the value at three standard deviations above or below the mean. This thresholding only pertains to the choropleth maps and does not affect the estimates for Massachusetts.

We see stark differences in the spatial distribution of ZCTA-level effects for the different subscales (Fig. 2a–f). For example, in the overall WBI, physical, financial, and community subscales, the spatial effect estimate for ZCTAs in Nantucket Island are the highest after adjusting for individual-level covariates. That is, after controlling for the set of individual demographic characteristics, we expect an about 22 unit increase in overall WBI due to residing in Nantucket ZCTA 02554. These ZCTA-level effects capture both observable and unobservable qualities of a ZCTA that can influence an individual’s well-being. Note that traveling from Nantucket to Martha’s Vineyard or the contiguous Massachusetts can only be accomplished by ferry and the ferry rides take longer than 30 minutes. As a result, the three ZCTAs on Nantucket are only neighbors with each other. Many ZCTAs in western Massachusetts yield positive ZCTA effects for the overall scale and each of the five subscales. Conversely, ZCTAs congregated in central Massachusetts, especially those near and to the west of Worcester, as well some south of Boston generally have the lowest estimated spatial effects, indicating decreases in well-being due to belonging to those ZCTAs. For example, the town of Braintree to the south of Boston has the lowest financial ZCTA effect of $-10.2$. This means that on average, we expect an individual’s financial well-being is 10.2 units lower due to residing in ZCTA 02184, holding all the individual-level demographic characteristics constant.

We inspected the ZCTA effect estimates for ZCTAs with survey responses versus for those without. For overall WBI, we find that the range of ZCTA effects for ZCTAs without observations is $(-5.5, 20.8)$, whereas the range of ZCTA effects for ZCTAs with responses is $(-10.3, 22.2)$. There is a much larger range for ZCTAs with respondents and this is expected since spatial effect estimates for ZCTAs with no observations are solely a function of neighboring ZCTAs through the spatial smoothing incorporated within the model. There is only one ZCTA (02713), a group of islands northwest of Martha’s Vineyard, that has no observations and no neighbors (based on driving time) and thus our model is unable to estimate its ZCTA effect.

**Figure 3:**
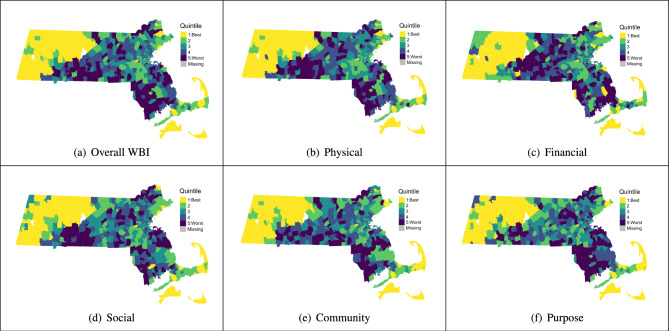
Choropleth maps of estimated ZCTA effects for overall WBI and the five subscales broken down into quintiles for Massachusetts.

We examined the estimated ZCTA effects broken down into quintiles for overall WBI and the five subscales (Fig. 3a–f). The majority of the top ranked ZCTAs across all subscales are in western Massachusetts, Cape Cod, Martha’s Vineyard, and Nantucket. Conversely, ZCTAs in the bottom quintile are predominantly situated in central and southeastern Massachusetts. Additionally, there seems to be larger clusters of similarly ranked ZCTAs in western Massachusetts compared to ZCTAs around Boston and its suburbs. One potential explanation for this is that since we are using driving times to inform our spatial neighborhoods, a 30-min drive in a rural location such as western Massachusetts will cover a larger area than a 30-min drive in an urban setting like Boston. Thus, ZCTAs in western Massachusetts are borrowing information from neighbors that are farther away (in terms of distance) than the neighbors of a ZCTA near Boston, thereby creating a larger smoothing effect. Additionally, there are more ZCTAs with no observations in western Massachusetts and the effect estimates for those ZCTAs are solely determined by spatial smoothing from neighboring ZCTAs.

### Georgia

Similar to Massachusetts, we find that education and income have large, positive, and statistically significant effects on overall WBI and each of the five subscales in Georgia (Table 3). In fact, most variables are statistically significant due to Georgia’s large sample size. Compared to the reference group of less than a high school education, we expect that having a post-graduate degree will lead to a 7.1 (95% CI: 6.0–8.3) unit increase in an individual’s overall WBI, holding all else constant. We also see large and statistically significant increases of at least 5.8 units for all subscales as well. Furthermore, we expect that on average, compared to the incomes less than $\$25,000$, having an income of at least $\$100,000$  will increase an individual’s overall WBI by about 8.7 (95% CI: 8.3–9.2) units, with a 21.5 (95% CI: 20.8–22.2) unit increase in the financial subscale, holding all else constant. An interesting finding is females, compared to males, have statistically lower well-being for the overall WBI and each of the five subscales. Those that are married, as compared to those that have never been married, have statistically significant increases in well-being for the social, community, and purpose subscales, with coefficient estimates of 7.4 (95% CI: 7.0–7.7), 4.4 (95% CI: 4.1–4.7), and 2.7 (95% CI: 2.3–3.0), respectively. Details on model fit are provided in Section 4 of the Supplementary Materials.

**Figure 4:**
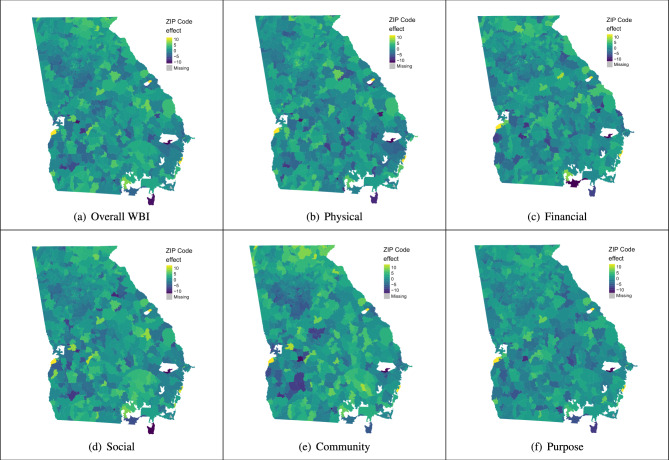
Choropleth maps of estimated ZCTA effects for overall WBI and the five subscales for Georgia. These ZCTA effect estimates represent the effect belonging to a ZCTA has on WBI and the five subscales after controlling for individual-level demographic covariates. To facilitate improved visualization, we threshold the estimated spatial effects exceeding three standard deviations from the mean and make them equal to the value at three standard deviations above or below the mean. This thresholding only pertains to the choropleth maps and does not affect the estimates for Georgia.

Although the spatial distribution of ZCTA effects in Georgia for overall WBI and the five subscales appears more homogeneous than that of Massachusetts, the standard deviation of ZCTA effects is roughly the same between the two states (Fig. 4a–f). We notice some general trends however, such as ZCTAs to the south of Atlanta as well as ones in southwestern Georgia having low ZCTA effects, especially in the community and purpose subscales. This suggests a decline in well-being due to belonging to those ZCTAs after adjusting for individual-level demographic characteristics. We find positive effects for ZCTAs in northern and southeastern parts of the state, indicating an improvement in well-being due to residing in those ZCTAs. For example, in the community subscale, ZCTA 30571, located in northern Georgia, has an effect of about 10.9. This means that compared to the average Georgia ZCTA, we expect a 10.9 unit increase in community well-being for individuals residing in ZCTA 30571, holding all the individual-level demographic characteristics constant. Lastly, it is important to note that part of Augusta, GA, captured by ZCTA 30905, fares best in overall WBI and almost all of the subscales. In each of the subscales in Fig. 4a–f, the ZCTA-level effect had to be trimmed in order to better visualize the spatial distribution of ZCTA effects.

We also examined the distribution of ZCTA effects between ZCTAs with observations and those without. The range of ZCTA effects for ZCTAs with observations is ($-18.6$, 25.1). The range of estimated effects for ZCTAs without observations is much larger ($-46.7$, 10.6), though it is largely driven by the presence of a couple extreme outlier ZCTAs with effect estimates $-41.0$ and $-46.7$. Interestingly, both of these outlier ZCTAs represent military bases (Moody AFB and Fort Stewart). Without the presence of these two ZCTAs, the range of ZCTA effects for ZCTAs without observations is ($-2.0, 10.6$). Note that all of these ZCTA effect estimates are the actual estimates (unlike the trimmed versions used in Fig. 4a–f). Additionally, there are only 26 ZCTAs with no observations in Georgia, compared to 91 for Massachusetts. Similar to Massachusetts, there is one ZCTA (31547) for which our model cannot generate a ZCTA effect because it has no observations and no neighbors.

**Figure 5:**
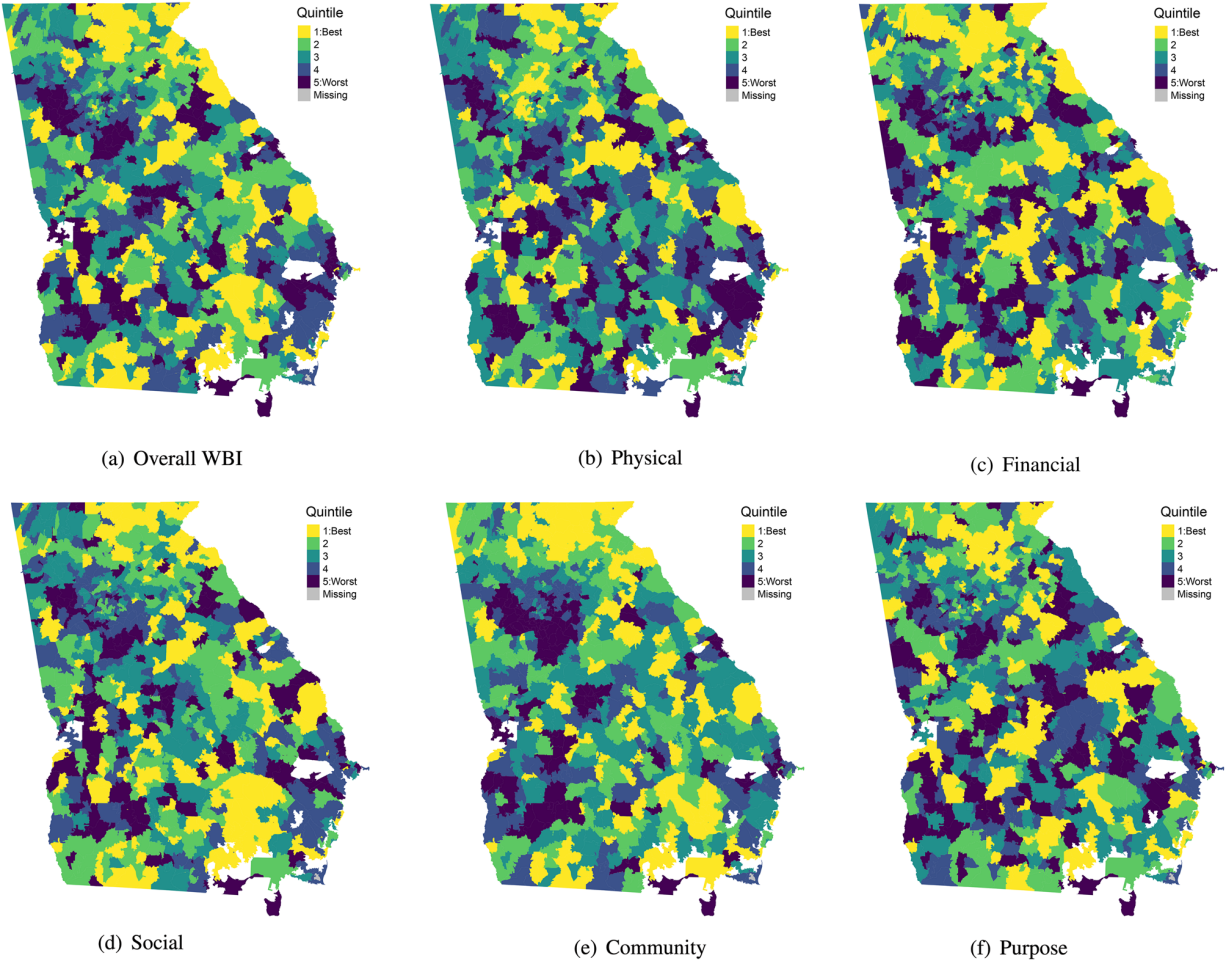
Choropleth maps of estimated ZCTA effects for overall WBI and the five subscales broken down into quintiles for Georgia.

ZCTA-level spatial effects broken down into quintiles also confirm that many of the top ranked ZCTAs are situated in northeastern Georgia (Fig. 5a–f). For the social, community, and purpose subscales, there are several ZCTAs in the southeast portion of the state that rank in the top quintile. Conversely, ZCTAs in the bottom quintile are predominantly located south of Atlanta, with some pockets occurring in southwestern Georgia as well. Additionally, we notice some larger pockets of similarly ranked ZCTAs in the northeast region of the state and also to the south of Atlanta, especially in the community subscale. In comparison to Massachusetts however, the size of clusters of similarly ranked ZCTA clusters appears smaller and this could be attributed to the fact that ZCTAs in Georgia, especially ones in the southern part of the state are larger and thus each ZCTA potentially has fewer neighbors, thereby diminishing the smoothing effect.

## Discussion

For these analyses we have used the Sharecare Well-Being Index and subscales, which are based on a comprehensive set of 52 items designed to provide an accurate measure of personal health and well-being across important domains^11^. Our analysis also further validates the idea that individuals interact outside of their municipal boundaries using resources and amenities from their neighboring ZCTAs. In shaping local policy, leaders may consult surrounding areas to better understand the breadth of residents’ lived experiences, and consider synergizing community improvement efforts beyond their own ZCTAs. As noted in this work, greater individual education and income is associated with higher individual report of well-being. It has been shown that individuals with better well-being live in environments with more available resources^15,16^, promoting health and well-being. We also found that Black individuals in Georgia, had lower overall WBI and in physical, financial and purpose subscales, which also perhaps points to some of the challenges certain racial communities face in terms of discrimination and/or access to resources^17,18^. Although beyond the scope of this paper, future research affords the opportunity to further examine and validate the associations between ZCTA-level social determinants of health and self-reported well-being to ultimately frame the association between one’s environment and their health.

### Spatial neighborhood and ZCTAs with zero responses

In this paper, we examined how ZCTAs affect community well-being. When evaluating effects of such small geographical units, spatial smoothing is useful in borrowing information from the neighboring entities to more accurately capture the effect of a ZCTA on its resident’s health and well-being. It enables enhanced estimation of well-being and has important implications for both researchers and local policymakers. It can improve small-area estimation of health outcomes and other community measures from sparse sample sizes, consequently allowing decision makers to be equipped with the necessary information to implement policy interventions. Our model uses a graph Laplacian matrix to capture the neighborhood structure that allows for leveraging information from neighboring ZCTAs to quantify ZCTA spatial effects. The main advantage to using this model is that even for ZCTAs with no observations, the model is still able to obtain ZCTA effect estimates, given that the ZCTA has a neighbor with at least one observation. Additionally, estimations on ZCTA effects can be used to generate rankings of ZCTAs. These rankings represent the measureable and unmeasurable qualities of a ZCTA that contribute to an individual’s overall well-being and wellness along the five different subscales after adjusting for individual-level demographic characteristics. Thus, they quantify the expected improvement or deterioration of an individual’s well-being due to belonging in that ZCTA.

### Limitations

One of the main limitations of employing ZCTAs as the geographical unit of interest is that they are much smaller spatial units and the number of responses within each ZCTA is vastly reduced when compared to sample sizes at the state- or county-level. Since rankings are based on participants included in the data and not based on all the people living in a particular ZCTA, small sample sizes can lead to estimates with higher standard errors. Although our framework of incorporating spatial neighborhood structures will return estimates for ZCTAs with zero responses, for areas where all ZCTAs have small sample sizes, spatial effect estimates may be imprecise or extreme. For example, in the financial subscale, the spatial effect for ZCTA 30905 is 62.2. However, that ZCTA has only two responses leading to the estimates being heavily impacted by them. Additionally, the Massachusetts and Georgia surveys are based on a convenience sample and may not accurately reflect the demographic make-up of the spatial units of interest. In this study, the sample is predominantly white, well educated, and high income (see Table 1), which may limit our ability to understand the effect of ZCTA characteristics in underrepresented populations. Furthermore, the only predictors in our models are the seven different demographic features; this results in a large amount of heterogeneity and unexplained variance, as evidenced by the low adjusted $R^2$ values.

### Future directions

The overall well-being scores are bounded between 0 and 100. In future work, we plan to extend our framework to a beta regression model, which is often used for distributions with a bounded support. Currently, our model setup can be deployed on any single state in the United States and even for small regions of states. As a future direction we aim to develop scalable model estimation strategies that enable implementing the models for the entire country. To do this, we will consider employing nearest neighbor Gaussian processes^19^, that are based on approximate inference strategies making them highly scalable in such spatial model settings. We are working on extending our framework to include survey data from multiple years to study the changes in ZCTA-level well-being measures over time while incorporating both spatial and temporal characteristics using Bayesian models. We are also working on extending our framework to determine whether communities with similar social determinants of health share similar individual reports of well-being. Thus, our goal is to harness our findings to better estimate ZCTA- and community-level well-being.

## Methods

### ZCTA neighborhood based on driving time

We defined neighbors using driving times between ZCTA population centroids. Population centroids were identified by calculating the population-weighted mean latitude and longitude for all of the high-resolution population point estimates from the GHS-POP R2023A dataset^20^ (updated to the 2021 ACS 5-year population estimates) that fell within each ZCTA^21^. These population estimates are provided at a resolution of approximately 100 meters; we substituted block geographic centroids in particularly densely populated areas in which block centroids would provide a higher resolution than what is provided by GHS-POP R2023A. ZCTAs with 0 population as of the 2020 Census were not given a population centroid due to the lack of population; this lead to 2/539 (0.4%) ZCTAs in Massachusetts and 3/751 (0.4%) ZCTAs in Georgia to be omitted in the construction of the graph Laplacian matrix, but no observations were dropped.

We define two ZCTAs to be neighbors if the driving time between their centroids is $\le 30$ minutes. We chose a 30-min cutoff because the median travel time to a cancer care site for Medicare beneficiaries aged 65+ in the southeastern USA is about 32 min^22^ and the average one-way commute time in the United States was about 28 min in 2019^23^. This gives us a reasonable estimate of how long people are willing to travel to seek medical care and employment. However, driving time cutoffs can be chosen to align with the conditions of a particular state or region under consideration, and such differences in what constitutes a *long drive* likely vary regionally. We conducted a sensitivity analysis with a driving time cutoff of 60 minutes and found that regression coefficients remained fairly consistent as did ZCTA quintile rankings; more details can be found in the Section 5 of the Supplementary Materials.

Driving times between ZCTA population centroids were calculated using OpenRouteService, which is an open-source geographic information system (GIS) program that can perform several types of routing calculations, including directions, geocoding, and isochrones for various modes of travel^24^. For this analysis, we used the openrouteservice R package to calculate drive-time matrices between all of the 2021 ZCTA population centroids for both Massachusetts and Georgia^25^. Using driving times to inform spatial neighborhoods is more meaningful than using Euclidean distances because it more accurately reflects practical travel times and distances between ZCTAs and de facto accounts for things like population density and road types. This is particularly important for standardizing our definition of spatial adjacency in urban vs. rural locations. For example, driving 30 minutes in a densely populated city will cover far fewer miles than driving 30 minutes in a rural area. Another advantage is that neighbors will not be limited to only adjacent ZCTAs or ZCTAs that share a boundary, which more appropriately reflects how people often cross multiple boundaries to seek out healthcare, food, social events, and other resources. Lastly, all maps of Massachusetts and Georgia in this paper and the Supplementary Materials were created using the tmap and tmaptools R packages^26^.

### Model setup

Let us assume that we have *S* ZCTAs with $n_s$ survey participants from each ZCTA $s = 1,\ldots ,S$, and $n = \sum _{s=1}^S n_s$ be the total number of observations. Let $y_{si}$ denote the well-being score (overall WBI or subscales) for participant *i* from ZCTA *s*. Consider the model

1 $$\begin{aligned} y_{si} = {\textbf{x}}_{si}^T\varvec{\beta } + \alpha _s + \epsilon _{si}, \end{aligned}$$

where $\textbf{x}_{\textbf{si}} = (x_{si,1},\ldots ,x_{si,p})^T$ are *p* covariates for the participant *i* from ZCTA *s*, $\varvec{\beta } = (\beta _1,\ldots ,\beta _p)^T$ are the corresponding regression coefficients, $\alpha _s$ is the intercept for ZCTA *s*, and $\epsilon _{si} \sim N(0,\sigma ^2)$ is the error term. Note that $\alpha _s$ is the ZCTA-level parameter that captures the effect of ZCTA *s* on the well-being scores $y_{si}$. The model in Equation (1) can also be written in matrix notation as

2 $$\begin{aligned} {\textbf{y}} = X\beta + \textbf{Z}\varvec{\alpha } + \varvec{\epsilon }, \end{aligned}$$

where ${\textbf{Z}}$ is a matrix with 0/1 entries that indicates the ZCTA each observation belongs to.

### Defining neighborhood using graph Laplacian matrix

We incorporate spatial information about the ZCTA through the ZCTA spatial effect parameter $\alpha _s$ and assume that the contribution of a ZCTA to the individual WBIs is correlated with its neighboring ZCTAs. We incorporate this spatial dependence assumption by defining driving time based ZCTA neighborhood structure (as described above) and including it into the model via a graph structure-based penalty term that involves a graph Laplacian matrix. A graph Laplacian matrix is defined as $L = D-A \in {\mathbb {R}}^{S \times S}$, where *A* is the (weighted) adjacency matrix and *D* is the corresponding degree matrix. The choice of these matrices jointly specify the desired spatial neighborhood structure. Further details on the construction of these matrices are provided in Section 2 of the Supplementary Materials. Note that a graph Laplacian matrix allows for borrowing information from neighboring ZCTAs, in addition to using individual-level data from that ZCTA, to estimate the ZCTA-level effect on well-being. Graph Laplacian methods have been used in different applications, especially in computer science. For example, a graph Laplacian regularization was used in a sparse representation model for image classification since neighboring pixels typically have similar representation coefficients^27^; facial recognition applications applied a Laplacian penalty to induce spatial smoothing constraints as neighboring pixels are correlated^28^. Note that, neighboring ZCTAs here refers to ZCTAs whose population centroids are within a 30-minute driving time of a target ZCTA’s population centroid. In the simulations outlined in the Supplementary Materials, we used a 25-mile Euclidean distance threshold between ZIP Code centroids rather than driving times. Although these two ways of defining neighborhood are different, our method is applicable in both cases and is flexible for other neighborhood definitions as well. Additionally, in both definitions of neighborhood (driving time and Euclidean distance), neighboring ZCTAs do not necessarily have to share a geographic boundary.

### Estimation of model parameters

To estimate the model parameters in Eq. (2), we consider the objective function ${\mathscr {L}}(\varvec{\beta },\varvec{\alpha }) = ({\textbf{y}} - X\varvec{\beta } - {\textbf{Z}}\varvec{\alpha })^T({\textbf{y}} - X\varvec{\beta } - {\textbf{Z}}\varvec{\alpha }) + \lambda \varvec{\alpha }^T L\varvec{\alpha }$. Here, the regularization term $\varvec{\alpha }^T L\varvec{\alpha }$, where *L* is the graph Laplacian matrix, incorporates the neighborhood structure between the ZCTAs. After some simple algebra we can see that $\varvec{\alpha }^T L\varvec{\alpha } = \sum _{s}\sum _{s' \in {\mathcal {N}}(s)} (\alpha _s - \alpha _{s'})^2$ where ${\mathcal {N}}(s)$ is the set of neighboring ZCTAs of the ZCTA *s*. These types of penalties have been previously proposed both in the linear and generalized linear mixed model frameworks in various applications^10,29,30^. The minimizer of ${\mathcal {L}}(\varvec{\beta },\varvec{\alpha })$ cannot be obtained in practice as the graph Laplacian matrix is singular by construction. For numerical stability, the usual strategy is to add a small positive constant to the diagonal of the graph Laplacian and update the penalty as $\lambda \varvec{\alpha }^T (L+\gamma I)\varvec{\alpha }$ where $\gamma > 0$. The updated penalty is same as including an additional ridge penalization, that is, $\lambda \varvec{\alpha }^T (L+\gamma I)\varvec{\alpha } = \lambda \varvec{\alpha }^T L\varvec{\alpha } + \lambda \gamma \sum _s \varvec{\alpha }_s^2$. Hence, the updated objective function is given as

3 $$\begin{aligned} {\mathscr {L}}(\varvec{\beta },\varvec{\alpha }) = ({\textbf{y}} - X\varvec{\beta } - {\textbf{Z}} \varvec{\alpha })^T({\textbf{y}} - X\varvec{\beta } - {\textbf{Z}}\varvec{\alpha }) + \lambda \varvec{\alpha }^T (L+\gamma I)\varvec{\alpha }. \end{aligned}$$

We have a closed-form solution for this optimization problem, and hence, we do not need to rely on numerical approaches to estimate the model parameters. The minimizer for this objective function is given by

4 $$\begin{aligned} \begin{bmatrix} \hat{\varvec{\beta }} \\ \hat{\varvec{\alpha }} \end{bmatrix} = ({\tilde{X}}^T{\tilde{X}} + M)^{-1} {\tilde{X}}^T{\textbf{y}}, \text { where } {\tilde{X}} = \begin{bmatrix} X&Z \end{bmatrix} \text { and } M = \begin{bmatrix} 0_{p \times p} &{} 0_{p \times S} \\ 0_{S \times p} &{} \lambda L + \lambda \gamma I_S \end{bmatrix}. \end{aligned}$$

Here, $\lambda$ and $\gamma$ are tuning parameters that can be estimated using cross-validation. This is accomplished through a grid search approach, in which a $(\lambda , \gamma )$ pair that minimizes the root mean squared error is selected. Once we have parameter estimates for ${{\hat{\alpha }}}$ and ${{\hat{\beta }}}$, we can predict well-being scores for new observations using ${\hat{y}}_{si} = {\textbf{x}}_{si}^T\hat{\varvec{\beta }} + {\hat{\alpha _s}}$, where ${\hat{y}}_{si}$ is the predicted well-being score for individual *i* belonging to ZCTA *s*, ${\textbf{x}}_{si}^T$ is a set of covariates for the new individual, $\hat{\varvec{\beta }}$ are the estimated regression coefficients, and ${\hat{\alpha _s}}$ is the estimated spatial effect of ZCTA *s*.

### Interpretation of the estimated ZCTA spatial effect

In our model setup, $y_{si} = {\textbf{x}}_{si}^T\varvec{\beta } + \alpha _s + \epsilon _{si}$, individual well-being score, $y_{si}$, increases as the ZCTA-level intercept $\alpha _s$ increases. That is, higher values of $\alpha _s$ indicate improvement in well-being measures for individuals belonging to that ZCTA. Note that $\alpha _s = \alpha _0 + {{\tilde{\alpha }}}_s$, where $\alpha _0$ is the overall intercept, i.e., average well-being across all observations in all ZCTAs, and ${{\tilde{\alpha }}}_s$ is the deviation from that (with a constraint that $\sum _s{{\tilde{\alpha }}}_s = 0$) for observations in ZCTA *s*. The estimate for ${\tilde{\alpha }}_s$ represents increase or decrease in a individual’s well-being score due to belonging to ZCTA *s*, with positive values indicating an improvement of well-being and vice-versa. Estimates for $\alpha _s$ (equivalently ${\tilde{\alpha }}_s$) can be used to rank ZCTAs as it determines how ZCTA characteristics are affecting individual well-being.

### Advantages of incorporating spatial information

There are several advantages of incorporating spatial information using a graph Laplacian matrix to evaluate ZCTA-level effects. First, neighbors of a ZCTA are determined based on driving times between their centroids and this information is included in the model via a truncated Gaussian spatial kernel. That is, as the driving time between ZCTAs increases, the level of influence of those neighboring ZCTAs decreases. ZCTAs closer to each other (in terms of driving time) have a higher spatial dependence in the effect estimates. ZCTAs with driving times between them greater than a certain threshold are not considered neighbors and thus do not directly influence each other’s estimates. Second, for those ZCTAs with responses, the estimates of spatial effects are informed both by responses within that ZCTA as well as information borrowed from its neighboring ZCTAs. Third, for ZCTAs with no responses, the estimates of spatial effects are informed by only the information borrowed from neighboring ZCTAs. This means that the only situations in which ZCTA-level spatial effects can not be estimated in our modeling framework is when a ZCTA with no responses (i) has no neighbors within the driving time threshold, or (ii) none of its neighbors have any responses as well. Note that, when working with granular geographical units such as ZCTAs, the number of responses are fewer, which makes a spatial smoothing-based approach more appealing and useful.

### Approval, accordance, and informed consent

This study involving human subjects was approved by Boston University Medical Campus Institutional Review Board and conducted in accordance with the relevant guidelines and regulations. All participants signed an informed consent form prior to enrollment. All methods were carried out in accordance with relevant guidelines and regulations.

### Supplementary Information

Supplementary Information.

## Data Availability

The data that support the findings of this study are available from Sharecare and BU School of Public Health, but restrictions apply to the availability of these data, and so are not publicly available. Data can be requested from the authors with permissions from both Sharecare and the BU School of Public Health.
