# Supplementary material for: Modeling health and well-being measures using ZIP code spatial neighborhood patterns
Source: Sci Rep. 2024 Apr 22;14:9180. doi: 10.1038/s41598-024-58157-w (PMC11035567; doi:10.1038/s41598-024-58157-w)
Supplement: Supplementary file 1 — Supplementary Information. [file 41598_2024_58157_MOESM1_ESM.pdf]

Supplement to “Modeling health and well-being measures using ZIP Code spatial neighborhood patterns”

Abhi Jain[1], Michael LaValley[1], Kimberly Dukes[1,*], Kevin Lane[2], Michael Winter[3], Keith R. Spangler[2], Nina Cesare[3], Biqi Wang[1,4], Michael Rickles[5], and Shariq Mohammed[1,6,*]

[1]Department of Biostatistics, Boston University School of Public Health, Boston, 02118, USA
[2]Department of Environmental Health, Boston University School of Public Health, Boston, 02118, USA
[3]Biostatistics and Epidemiology Data Analytics Center, Boston University School of Public Health, Boston, 02118, USA
[4]Department of Medicine, University of Massachusetts Chan Medical School, Worcester, 01655, USA
[5]Sharecare, Research and Outcomes, Atlanta, 30305, USA
[6]Rafik B. Hariri Institute for Computing and Computational Science and Engineering, Boston University, Boston, 02215, USA
[*]Corresponding authors emails: shariqm@bu.edu, dukeska@bu.edu

ABSTRACT

We present additional results from exploratory data analysis on the Sharecare data for Massachusetts and Georgia. Specifically, we examine additional summary statistics for overall WBI and the five subscales as well as the spatial distribution of mean scores for those variables at the ZCTA level. We also discuss details on the construction of the graph Laplacian matrix and estimation of standard errors for regression coefficient and ZCTA effect estimates. Additionally, we perform simulations under various sample size and noise settings. We test our model on four different spatial patterns and report several model performance metrics. Our model is able to successfully capture these spatial patterns under each sample size and noise setting. We end by presenting some model fit metrics for our real data analysis on Massachusetts and Georgia.

1 Data

Here we present exploratory data analysis of the overall Well-Being Index (Overall WBI) and well-being across the five subscales (Physical, Financial, Social, Community, and Purpose) for ZCTAs across Massachusetts and Georgia. We find that ZCTAs around Boston as well as some in Western Massachusetts and Cape Code have the highest average scores for overall WBI and the five subscales (Supplementary Figures S1(a) - S1(f)). Generally, we find lower average scores in ZCTAs in southeastern Massachusetts and also west of Worcester, especially in the Financial subscale. Note that there are several ZCTAs with zero observations in western Massachusetts. In Georgia, we find ZCTAs in northern Georgia have the highest average scores. ZCTAs south of Atlanta and also southeastern Georgia having the lowest scores across overall WBI and most of the subscales, especially the financial, community, and purpose subscales (Supplementary Figures S2(a) - S2(f)).

Supplementary Table S1 shows more comprehensive statistics for average WBI and subscales across ZCTAs in the US, Massachusetts, and Georgia. We can see that Georgia generally performs reasonably well (based solely on the available well-being scores) across all subscales, with averages higher than both Massachusetts and the United States as a whole. Additionally, we also notice that the standard deviation for Georgia is lower that that of the US and Massachusetts, illustration a more homogeneous distribution across ZCTAs.

2 Methods

Construction of the graph Laplacian matrix. Note that the regularization term in our model involves a graph Laplacian matrix defined as $L = D - A \in \mathbb{R}^{S \times S}$, where A is the (weighted) adjacency matrix and D is the corresponding degree matrix. The adjacency matrix weights are calculated as $A_{ss'} = exp\left(\frac{-\delta^2_{ss'}}{2\omega^2}\right)$, where $\delta_{ss'}$ is the driving time between the population centroids of ZCTA s and s', and ω is the driving time cutoff (30-minutes in our case). Thus, ZCTAs whose population centroids are more than ω minutes away are not considered neighbors and thus their weight is equal to zero. Additionally, the degree matrix is

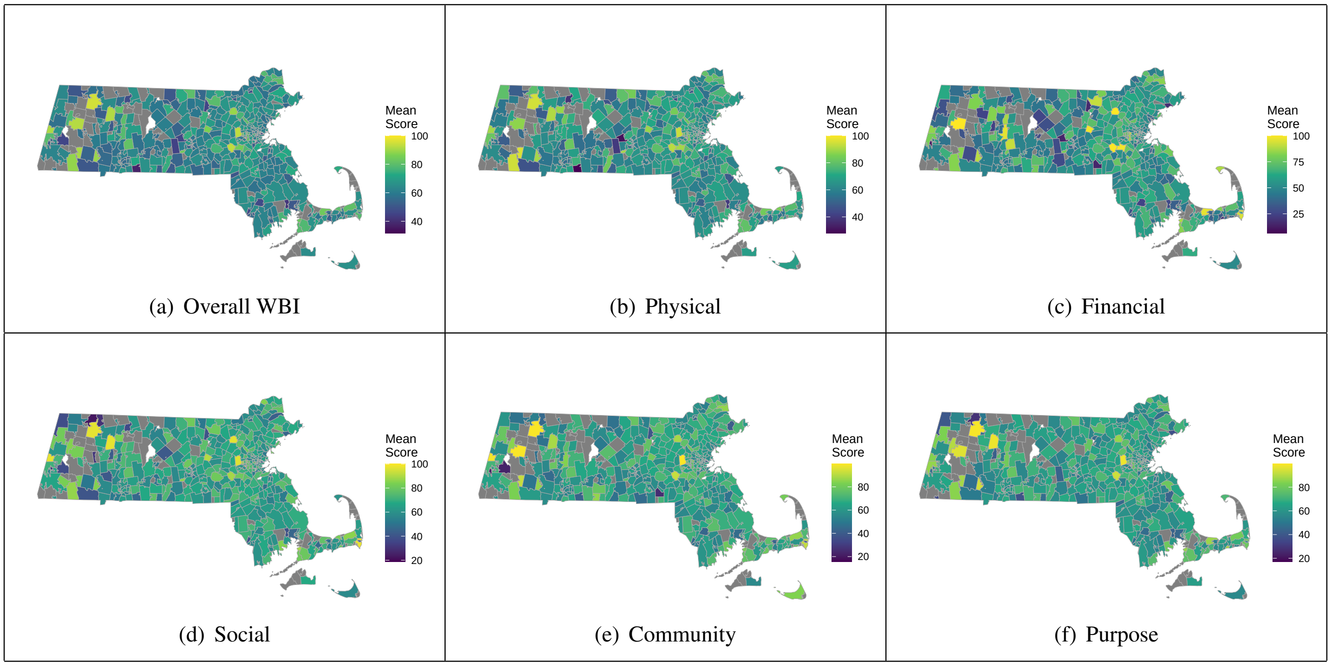

Supplementary Figure S1. Average values of overall WBI and the five subscales by ZCTA for Massachusetts. Gray ZCTAs are ones with zero observations. All maps of Massachusetts and Georgia in the Supplementary Materials were created using the tmap and tmaptools R packages.[1]

diagonal and is defined as $D_{ss} = \sum_{s'=1}^{S} A_{ss'}$ and $D_{ss'} = 0$, which corresponds to the out-degree degree matrix. Diagonal entries in the out-degree degree matrix represent the total driving time to travel to all other ZCTAs that are defined as neighbors, which makes sense in this analysis since we are interested in how individuals residing in a particular ZCTA travel to neighboring ZCTAs to access resources and amenities.

Estimation of standard errors. Recall that in ordinary least squares (excluding the spatial regularization term), $\hat{\beta}_{OLS} = (\tilde{X}^T\tilde{X})^{-1}\tilde{X}^T\mathbf{y}$ and $var(\hat{\beta}_{OLS}) = \sigma^2(\tilde{X}^T\tilde{X})^{-1}$. Since our model is simultaneously estimating $\hat{\beta}$ and $\hat{\alpha}$, let $\hat{\theta} = \begin{bmatrix}\hat{\beta}\\ \hat{\alpha}\end{bmatrix}$. We have

$$\begin{aligned}\hat{\theta} &= (\tilde{X}^T\tilde{X}+M)^{-1}\tilde{X}^T\mathbf{y} &= (\tilde{X}^T\tilde{X}+M)^{-1}(\tilde{X}^T\tilde{X})(\tilde{X}^T\tilde{X})^{-1}\tilde{X}^T\mathbf{y}\\ & &= (\tilde{X}^T\tilde{X}+M)^{-1}(\tilde{X}^T\tilde{X})\hat{\theta}_{OLS}\end{aligned}$$

Therefore, the variance of the estimates for our model with spatial regularization is given as

$$\begin{aligned}var(\hat{\theta}|\tilde{X}) &= (\tilde{X}^T\tilde{X}+M)^{-1}\tilde{X}^T\tilde{X}(var(\hat{\theta}_{OLS}|\tilde{X}))[(\tilde{X}^T\tilde{X}+M)^{-1}\tilde{X}^T\tilde{X}]^T\\ &= \sigma^2(\tilde{X}^T\tilde{X}+M)^{-1}\tilde{X}^T\tilde{X}(\tilde{X}^T\tilde{X}+M)^{-1}\end{aligned}$$

Standard errors are then calculated by taking the square root of the diagonal entries of the above variance-covariance matrix.

Non-contiguous ZCTAs ZCTAs can be non-contiguous[2] and to evaluate the effect of non-contiguous ZCTAs on driving times, we calculated the number of non-contiguous ZCTAs in Massachusetts and Georgia and the maximum distances between the non-contiguous parts of each ZCTA (Table S2). Overall, there are only 8 ZCTAs out of 539 (1.5%) in Massachusetts and 27 ZCTAs out of 751 (3.6%) in Georgia that are non-contiguous, and among those that are non-contiguous, 80% are separated by less than 1 kilometer. The farthest distance between any non-contiguous ZCTA parts in both states is just over 4 kilometers. In the context of our spatial relationship definition of a 30-minute driving time (and 60 minutes in the sensitivity analysis), the error introduced by non-contiguity of ZCTAs, if any, is very likely negligible. Moreover, we used population centroids to represent the starting/ending points of the driving distances irrespective of ZCTA contiguity; our results would be the same if the non-contiguous ZCTAs were connected with an unpopulated polygon.

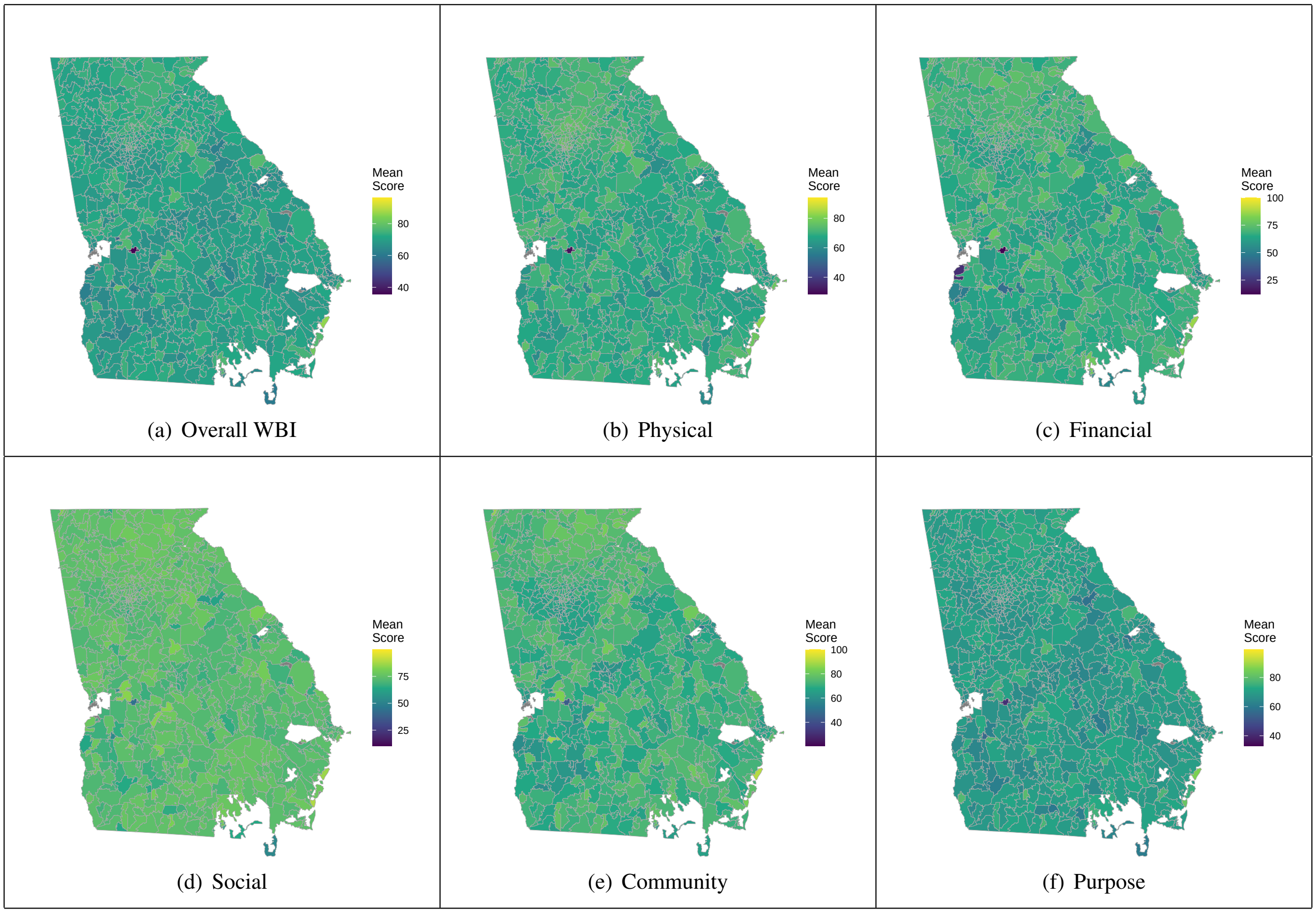

(a) Overall WBI (b) Physical (c) Financial (d) Social (e) Community (f) Purpose

Supplementary Figure S2. Average values of overall WBI and the five subscales by ZCTA for Georgia. Gray ZCTAs are ones with zero observations.

3 Simulation

We perform simulations to show that if spatial patterns exist at the ZIP Code level, then our model is able to successfully capture those spatial patterns under various sample size and noise settings. In this section, we evaluate the model's performance using a simulation study under different scenarios with Massachusetts as the reference state. Specifically, we will assess the performance of our model in terms of estimation, model fit, and prediction accuracy. In this section, we describe the overall simulation setup, construction of the design matrix, and the choice of ZIP Code spatial patterns. Additionally, note that for our simulations, we used ZIP Codes as the spatial unit instead of ZCTAs.

3.1 Simulation setup

We generate three explanatory variables for our analysis and attempt to mimic the explanatory variables in the MA WBI dataset. The first covariate X_1 is sampled from $\mathcal{N}(10, 9)$. The second and third covariates (X_2 and X_3) are assumed categorical: X_2 has three levels (1, 2, 3), simulated with probabilities 0.2, 0.5, and 0.3, respectively; X_3 has four levels (1, 2, 3, 4), simulated with probabilities 0.4, 0.3, 0.2, and 0.1, respectively. This yields a design matrix of six columns, with X_1 as the first column and the remaining columns as the following indicator variables: $\{I(X_2 = 2), I(X_2 = 3), I(X_3 = 2), I(X_3 = 3), I(X_3 = 4)\}$. The response y is simulated as:

$$y \sim \mathcal{N}(\alpha_0 + \beta_1 X_1 + \beta_2(X_2 = 2) + \beta_3(X_2 = 3) + \beta_4(X_3 = 2) + \beta_5(X_3 = 3) + \beta_6(X_3 = 4) + \tilde{\alpha}_s, \sigma^2),$$

where $\alpha_0 = 15$, $\beta_1 = 3$, $\beta_2 = 5$, and $\beta_3 = 10$, $\beta_4 = 6$, $\beta_5 = -4$, and $\beta_6 = 2$. Here, $\tilde{\alpha}_s$ is the spatial deviation corresponding to the ZIP Code (obtained as described below) assigned to the observation and $\alpha_s = (\alpha_0 + \tilde{\alpha}_s)$ represents the overall spatial effect. We perform simulations under various sample size (N) and noise (σ) settings: $N = 1000$, 5000, and 10000; and noise $\sigma = 5$, 10, and 20.

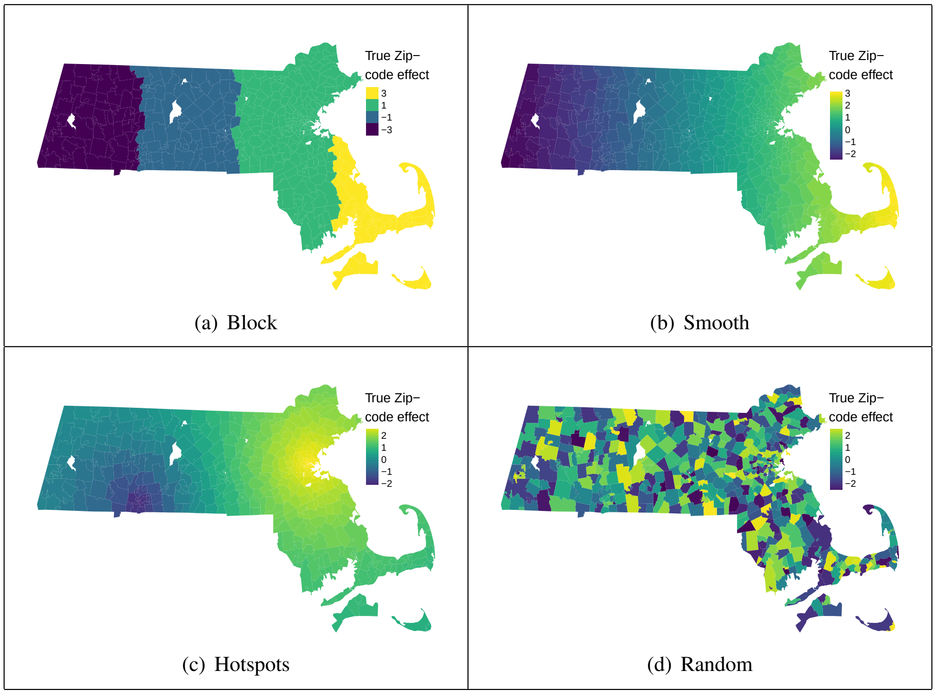

(a) Block (b) Smooth (c) Hotspots (d) Random

Supplementary Figure S3. Choropleth maps of the true ZIP Code spatial effects for each of the four spatial patterns we consider in the simulation study.

We assign these N observations to different ZIP Codes as follows. We obtain N samples (with replacement) of ZIP Codes from the MA WBI dataset in order to mimic the representation of MA ZIP Codes. Massachusetts has 536 ZIP Codes of which only 447 are included in the MA WBI dataset. That is, 89 ZIP Codes did not have any responses, hence, these ZIP Codes will never be sampled. Note that, since we are sampling ZIP Codes with replacement from the original MA data set, certain ZIP Codes will have low probability of being sampled in the simulated dataset, which could result in such ZIP Codes not being sampled due to random chance. Additionally, in this section, we use the Euclidean distance to inform the spatial neighborhoods with $\omega = 25$ miles as the cutoff. Although we fix $\omega = 25$ miles for the purposes of this simulation, ω can be assigned to examine how different distance thresholds effect estimation of regression coefficients and ZIP Code-level spatial effects. Note that we use driving time to inform spatial neighborhoods in the manuscript for real data analysis. Both approaches to define ZIP Code neighborhood (distance-based and driving time-based) can be used in practice. For the purpose of this simulation study, as a proof of concept, we used distance between ZIP Codes for the same, but it can readily be deployed with driving time-based neighborhood as well.

The ZIP Code-level spatial deviations $\tilde{\alpha}_s$ are assigned under four different spatial patterns[3]: *block*, *smooth*, *hotspots* (Boston and Springfield), and *random*. Supplementary Figures S3(a) - S3(d) illustrate these four spatial patterns. For the block structure, Massachusetts ZIP Codes are categorized into one of four vertical blocks determined by the longitudes of the ZIP Code centroids. The only exception is that the ZIP Codes north of Boston with a longitude greater than -70.84 are placed in Block 3 instead of Block 4 for spatial continuity of Block 4. Of the 536 ZIP Codes in MA, 81 are in Block 1, 107 are in Block 2, 271 are in Block 3, and 77 are in Block 4. The spatial effects for ZIP Codes are determined by the block they belongs to and are assigned as $-3, -1, 1$ and 3 for Blocks 1 to 4, respectively. In the smooth pattern, the spatial deviations steadily increase from $\tilde{\alpha}_s = -3$ to $\tilde{\alpha}_s = 3$ as we move from West to East, with Cape Cod, Martha's Vineyard, and Nantucket having the highest spatial deviation. In the hotspots pattern, we choose the cities of Boston and Springfield as hotspots. For the purpose of this simulation, we assign a positive spatial effect to Boston and negative spatial effect to Springfield. Additionally, the spatial effect of the ZIP Codes as we move further from Boston and Springfield, have a decreasing influence of the hotspots. Lastly, we consider a random spatial pattern in which spatial deviations are randomly sampled from a uniform distribution $\mathscr{U}\{-3, 3\}$.

We replicate the simulation 100 times for each combination of (N, σ). However, for brevity, we will present results from the median simulation setting with $(N = 5000, \sigma = 10)$. This setting is similar to the MA WBI data for the overall WBI scale in terms of N and sample size and standard deviationσ.

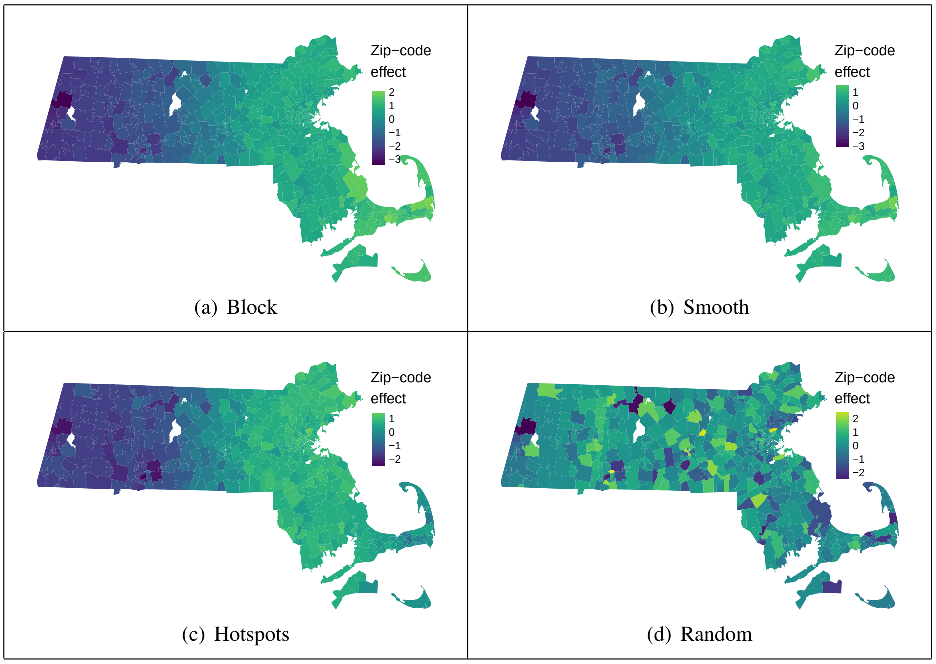

(a) Block (b) Smooth (c) Hotspots (d) Random

Supplementary Figure S4. Choropleth of estimated ZIP Code spatial effects from one replication under the $(N = 5000, \sigma = 10)$ simulation setting for each of the four different spatial patterns.

3.2 Model estimation

Overall, we find fairly accurate estimation of β coefficients. Most of the coefficient estimates are within 2-3% of the true value and estimation is consistent over all spatial patterns, as well as sample size and noise settings. Supplementary Figures S4(a) - S4(d) illustrates the ZIP Code-level spatial effect estimates from one replications of the $(N = 5000, \sigma = 10)$ simulation setting. For both the block and smooth spatial patterns, we observe the spatial effects increasing as we move from West to East, with the smooth pattern depicting a slightly more continuous increase. Additionally, for the hotspots pattern, we find that the lowest spatial effects are concentrated near Springfield and the highest spatial effects are concentrated near Boston. Lastly, for the random spatial pattern, we find no discernible pattern in the spatial distribution of $\hat{\tilde{\alpha}}_s$. Thus, for each spatial structure, the general pattern of estimated spatial effects mimics the structure of true spatial effects. Additionally, Supplementary Table S3 shows the Spearman correlation between $\tilde{\alpha}_s$ and $\hat{\tilde{\alpha}}_s$ for each simulation setting and spatial pattern. As expected, we observe that the correlation increases as sample size increases and noise decreases. We find that the smooth pattern has the highest correlation (random pattern has the lowest correlation) since the spatial smoothness assumption is aligned with the assumptions of the model.

Although the Spearman correlations between true and estimated spatial effects are high, there are some ZIP Codes with large discrepancies, especially in the block and hotspots patterns. Supplementary Figures S5(a) - S5(d) depict the spatial effect residuals $(\tilde{\alpha}_s - \hat{\tilde{\alpha}}_s)$ for ZIP Codes from one replication under the $(N = 5000, \sigma = 10)$ simulation setting in each of the four spatial patterns. In the block structure, we notice that the magnitude of the spatial effect residuals are highest at ZIP Codes near the border of the blocks. This is expected as true spatial effects sharply increase by two units as the blocks move from West to East. When observing the map of spatial effect residuals in the smooth and hotspot structures, we don't find any evident spatial pattern. The only major feature we notice is that ZIP Codes with the highest true spatial effect in terms of absolute value tend to have larger spatial effect residuals and this is due to attenuation of spatial effects from the model. Lastly, we find no discernible pattern in the distribution of residuals for the random structure.

One of the advantages of our model is that it returns estimates of spatial effects for ZIP Codes no responses due to the spatial neighborhood based penalty. Supplementary Figure S6 illustrates the median absolute value and interdecile range of spatial effect residuals for ZIP Codes that were missing and present in the data. For every sample size, noise, and pattern setting, we find that the magnitude of spatial effect residuals is larger for ZIP Codes with zero responses – but still reasonable given that those ZIP Codes do not have any observations.

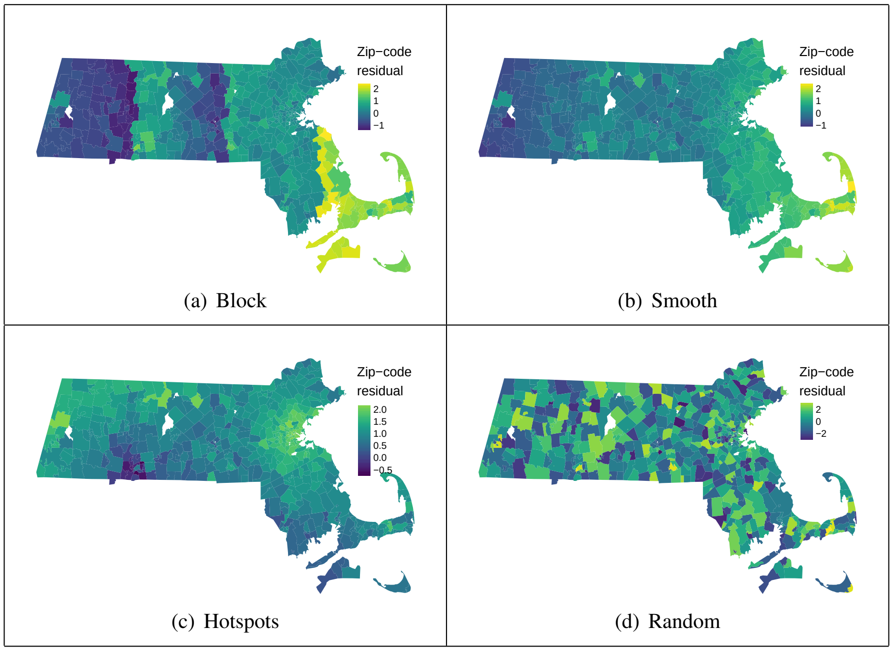

(a) Block (b) Smooth (c) Hotspots (d) Random

Supplementary Figure S5. Choropleth of estimated ZIP Code effect residuals from one replication under the setting $(N = 5000, \sigma = 10)$ simulation setting for each of the four different spatial patterns.

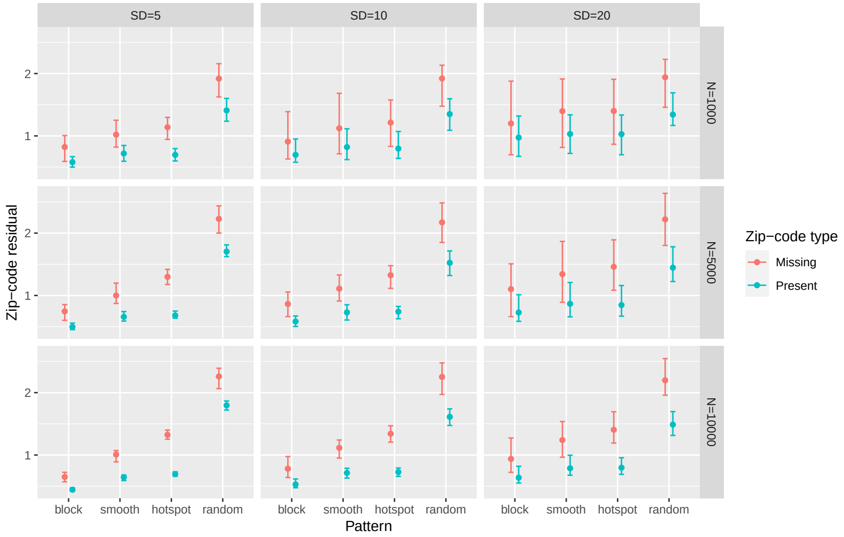

Supplementary Figure S6. Median and interdecile range of absolute value of spatial effect residuals by type of ZIP Code.

3.3 Model fit

In addition to evaluating our model's estimation of regression coefficients and spatial effects, we are also interested in evaluating the overall fit of the model and comparing it to that of a linear model and linear mixed model without spatial neighborhood structures. Supplementary Table S4 shows the average adjusted R^2 values for the linear model, linear mixed model and linear model with spatial effect for different noise settings and with the sample size fixed at $N = 5000$. We see that, for all patterns except the random spatial pattern, the linear model with spatial effect offers improvement in the adjusted R^2 over the linear mixed model, which has a higher adjusted R^2 than the linear model.

3.4 Prediction

Lastly, we test our model's ability to predict the outcome variable, i.e., the WBI scores. To facilitate prediction, we created a test dataset for each replication of each simulation setting. This test set was produced by randomly generating five observations

for each of Massachusetts's 536 ZIP Codes. Thus, each of the three covariates was randomly generated as described previously and the outcome variable y was simulated using the same set of true β coefficients and a spatial effect α_s that depends on the ZIP Code of the observation. Supplementary Table S5 shows the average RMSE of the test dataset. We can see that the average RMSE is roughly equal for each spatial pattern and also approximately equal to the noise. This means that the average difference between the predicted outcome and the true outcome is about one standard deviation.

Another metric we consider for prediction is the correlation between the true values in the test set and the predicted values. As we can see, the average correlation is highest for the low noise ($\sigma = 5$) setting with correlations around 0.9 for all spatial patterns and sample settings (Supplementary Table S6). In the medium ($\sigma = 10$) and high ($\sigma = 20$) noise settings, the correlation is around 0.72-0.73 and 0.46-0.49 for all spatial patterns and sample size settings, respectively.

4 Adjusted R^2 for real data analysis

We consider the adjusted R^2 for models with regularization[4]. The formula for the adjusted R^2 is:

$$R^2_{adj} = 1 - \frac{\sum_{i=1}^{N}(y-\hat{y})^2/(N-df(\lambda,\gamma))}{\sum_{i=1}^{N}(y-\bar{y})^2/(N-1)},$$

where $df(\lambda,\gamma)) = \text{trace}[\tilde{X}(\tilde{X}^T\tilde{X}+M)^{-1}\tilde{X}^T]$ and is the effective degrees of freedom.

We notice that for Massachusetts, the adjusted R^2 values are higher than that of Georgia for each subscale (Supplementary Table S7). Additionally, for both Massachusetts and Georgia, the financial subscale has the highest adjusted R^2 at 0.261 and 0.154, respectively and the lowest adjusted R^2 is in the purpose subscale, with values of 0.078 and 0.063, respectively.

5 Sensitivity analysis with 60-minute driving time threshold

In the data analysis for Massachusetts and Georgia presented in the article, we used a 30-minute driving time cutoff between ZCTA population centroids to inform our neighborhood structure. The driving-time cutoff however can be parameterized and different driving times may be more appropriate for certain geographies. Here, we perform a sensitivity analysis using a 60-minute driving time threshold for the state of Georgia. Firstly, we observe minimal differences in the coefficient estimates for most of our predictor variables, with noticeable differences in the magnitude observed only for *Education* and *Urban* (Table S8). Additionally, we can see that the overall spatial distribution of ZCTA effects is fairly similar between the 30-minute and 60-minute cutoffs (Supplementary Figures S7(a)-S7(b)). The top ranked ZCTAs are still around northeast Georgia and we still find many of the lowest ranked ZCTAs south of Atlanta and in the southwestern region of the state. The magnitude of the estimated ZCTA effects for the two driving time cutoffs are slightly different (as expected), that is, the estimated ZCTA effects with the 60-minute cutoff are generally smaller in magnitude compared to the 30-minute cutoff. This is expected since each ZCTA has more neighbors influencing its effect estimate with a 60-minute cutoff leading to a higher degree of spatial smoothing.

Although the ZCTA effects are estimated from smaller numbers of observations than the beta values and show some differences, the ZCTA quintile rankings are mostly consistent. Supplementary Table S9 shows the proportion of ZCTAs that belong to each quintile under a 30-minute and 60-minute driving time cutoff. Of ZCTAs ranked in the first quintile when using a 30-minute driving time cutoff, about 65% of them remain in the top quintile with a 60-minute driving time cutoff, and about 88% stay in the top two quintiles. Similarly, out of ZCTAs in the bottom quintile with a 30-minute driving time cutoff, 74% remain in the bottom quintile with a 60-minute driving time cutoff. In the middle quintiles, many of the ZCTA effect estimates are close to zero and small changes in these estimates may lead to a change in quintiles. We recognize that there are several aspects of the data and geography that affect ZCTA effect estimation, and careful consideration of an appropriate driving time threshold is required.

References

1. Tennekes, M. tmap: Thematic maps in R. *J. Stat. Softw.* **84**, 1–39, DOI: 10.18637/jss.v084.i06 (2018).
2. Grubesic, T. H. & Matisziw, T. C. On the use of ZIP codes and ZIP code tabulation areas (ZCTAs) for the spatial analysis of epidemiological data. *Int. journal health geographics* **5**, 1–15 (2006).
3. Halder, A., Mohammed, S., Chen, K. & Dey, D. K. Spatial tweedie exponential dispersion models: an application to insurance rate-making. *Scand. Actuar. J.* 1–20 (2021).
4. Hastie, T. *et al.* Linear Methods for Regression. *The Elem. Stat. Learn. Data Mining, Inference, Predict.* 43–99 (2009).

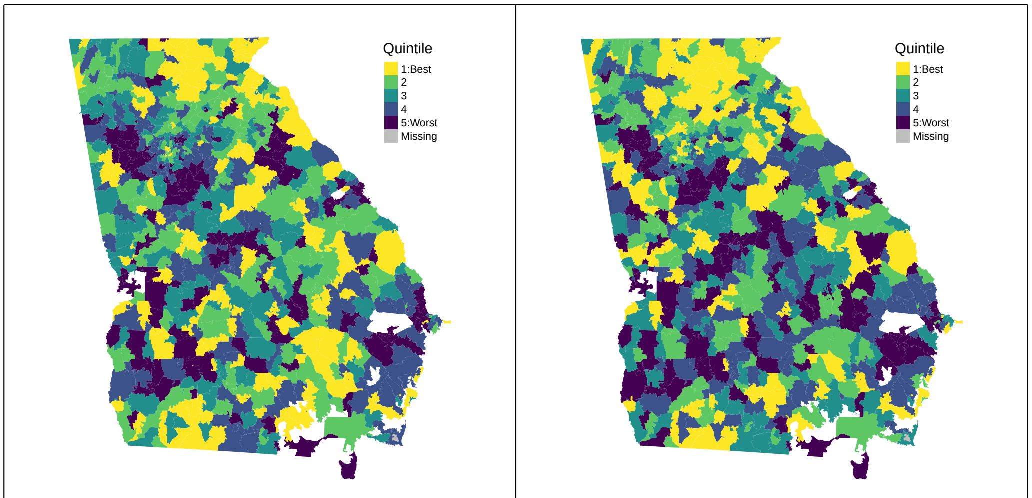

(a) 30-minute (b) 60-minute

Supplementary Figure S7. Choropleth maps of estimated ZCTA effects for overall WBI broken down into quintiles for Georgia using a 30-minute driving time cutoff versus a 60-minute driving time cutoff.

Supplementary Table S1. Summary statistics of overall WBI and five subscales for the entire United States, Massachusetts (MA), and Georgia (GA). The sample size for the US is 495,783 while the sample sizes for MA and GA are 4,443 and 116,808, respectively.

Index/State	Mean	SD	Median	IQR
Overall WBI				
US	68.18	15.67	69.40	21.95
MA	63.24	16.39	63.78	22.94
GA	70.68	14.93	71.84	21.03
Physical				
US	68.81	16.96	70.95	24.00
MA	64.83	18.28	66.82	26.21
GA	70.02	16.27	71.79	23.08
Financial				
US	64.13	24.70	66.97	37.61
MA	54.41	26.12	55.13	41.66
GA	66.85	23.48	69.87	35.34
Social				
US	69.65	19.46	72.33	27.72
MA	64.20	19.86	65.93	26.38
GA	73.31	18.29	75.95	25.69
Community				
US	66.51	19.12	68.17	26.78
MA	61.95	19.65	63.06	27.47
GA	70.21	18.14	72.14	25.71
Physical				
US	68.81	16.96	70.95	24.00
MA	64.83	18.28	66.82	26.21
GA	70.02	16.27	71.79	23.08

Supplementary Table S2. Five-number summary of maximum distances (kilometers) between non-contiguous ZCTA polygons for Massachusetts (MA) and Georgia (GA):

State	Minimum	25th percentile	Median	75th percentile	Maximum
MA	0.043	0.076	0.114	0.225	1.046
GA	0.025	0.167	0.569	0.931	4.077

Supplementary Table S3. Spearman correlations between true and estimated ZIP Code spatial effects.

Pattern	Block			Smooth			Hotspots			Random		
N	1,000	5,000	10,000	1,000	5,000	10,000	1,000	5,000	10,000	1,000	5,000	10,000
$\sigma = 5$	0.926	0.926	0.926	0.992	0.997	0.998	0.947	0.965	0.970	0.791	0.868	0.887
$\sigma = 10$	0.925	0.926	0.926	0.988	0.995	0.996	0.909	0.952	0.960	0.547	0.817	0.849
$\sigma = 20$	0.924	0.925	0.926	0.968	0.987	0.993	0.830	0.921	0.941	0.381	0.681	0.770

Supplementary Table S4. Average Adjusted R^2 for each model under different spatial patterns and noise settings. The sample size setting is fixed at $N = 5000$.

Pattern	Block			Smooth			Hotspots			Random		
σ	5	10	20	5	10	20	5	10	20	5	10	20
OLS	0.798	0.511	0.209	0.803	0.513	0.210	0.801	0.513	0.210	0.792	0.509	0.209
Linear Mixed	0.812	0.520	0.213	0.811	0.518	0.212	0.811	0.520	0.212	0.813	0.523	0.214
Linear Spatial	0.813	0.523	0.216	0.813	0.521	0.214	0.812	0.523	0.215	0.814	0.522	0.213

Supplementary Table S5. Average RMSE of test dataset.

Pattern	Block			Smooth			Hotspots			Random		
N	1,000	5,000	10,000	1,000	5,000	10,000	1,000	5,000	10,000	1,000	5,000	10,000
$\sigma = 5$	5.10	5.05	5.03	5.07	5.02	5.01	5.06	5.02	5.02	5.30	5.20	5.15
$\sigma = 10$	10.08	10.03	10.03	10.06	10.02	10.02	10.05	10.02	10.02	10.18	10.13	10.12
$\sigma = 20$	20.12	20.03	20.01	20.10	20.02	20.00	20.10	20.01	20.00	20.15	20.08	20.06

Supplementary Table S6. Average correlation between y_{si} and $\hat{y_{si}}$ in the dataset for all simulation settings and spatial patterns.

Pattern	Block			Smooth			Hotspots			Random		
N	1,000	5,000	10,000	1,000	5,000	10,000	1,000	5,000	10,000	1,000	5,000	10,000
$\sigma = 5$	0.91	0.90	0.90	0.91	0.90	0.90	0.91	0.90	0.90	0.91	0.91	0.91
$\sigma = 10$	0.73	0.73	0.73	0.73	0.73	0.72	0.73	0.73	0.72	0.72	0.72	0.73
$\sigma = 20$	0.49	0.48	0.48	0.48	0.47	0.47	0.47	0.47	0.47	0.46	0.46	0.47

Supplementary Table S7. Adjusted R^2 for both Massachusetts and Georgia for overall WBI and all subscales.

State/Subscale	Overall WBI	Physical	Financial	Social	Community	Purpose
Massachusetts	0.174	0.139	0.256	0.104	0.119	0.081
Georgia	0.107	0.065	0.154	0.087	0.097	0.063

Supplementary Table S8. Regression results for Overall WBI for Georgia using a 30-minute vs. 60-minute driving time cutoff. Reference groups for the categorical variables are given in parentheses next to the variable name. Coefficient estimates in bold are statistically significant at the 5% level and 95% confidence intervals are in parentheses.

	30-minute cutoff	*60-minute cutoff*
Gender (ref: Male)		
Female	−**1.1** (−1.3, -0.9)	−**1.1** (-1.3, −0.9)
Age (ref: 18-29)		
Age: 30-44	**0.4** (0.0, 0.8)	0.3 (−0.1, 0.7)
Age: 45-64	**3.7** (3.3, 4.0)	**3.6** (3.2, 3.9)
Age: 65+	**8.8** (8.3, 9.4)	**8.7** (8.2, 9.3)
Race (ref: White)		
Black	−**0.8** (−1.0, −0.5)	−**0.9** (−1.1, −0.6)
Hispanic/Latino	**1.2** (0.7, 1.7)	**1.1** (0.7, 1.6)
Asian	**4.0** (3.4, 4.6)	**4.0** (3.4, 4.6)
Other Race	0.1 (−0.6, 0.8)	0.1 (−0.6, 0.8)
Marital (ref: Never Married)		
Married	**3.2** (2.9, 3.5)	**3.1** (2.8, 3.4)
Other Marital Status	**1.5** (1.2, 1.9)	**1.5** (1.2, 1.8)
Education (ref: $<$ High School)		
High School	**4.5** (3.4, 5.6)	**2.8** (1.6, 3.9)
College	**5.4** (4.2, 6.5)	**3.7** (2.5, 4.8)
Post-Graduate	**7.1** (6.0, 8.3)	**5.4** (4.3, 6.6)
Income (ref: $<$ 25K)		
Income: 25-50K	**2.5** (2.1, 3.0)	**2.4** (1.9, 2.8)
Income: 50-100K	**5.4** (5.0, 5.9)	**5.2** (4.8, 5.7)
Income: 100K+	**8.7** (8.3, 9.2)	**8.6** (8.1, 9.0)
Urban (ref: Rural)		
Urban	**1.9** (1.1, 2.6)	0.2 (−0.2, 0.5)

Supplementary Table S9. Proportion of ZCTAs in each quintile under a 30-minute driving and 60-minute driving-time threshold.

30-min/60-min	1:Best	2	3	4	5:Worst
1:Best	0.65	0.23	0.08	0.02	0.02
2	0.32	0.35	0.28	0.05	0.01
3	0.03	0.35	0.30	0.28	0.04
4	0.00	0.06	0.30	0.46	0.19
5:Worst	0.00	0.01	0.05	0.20	0.74
